# The epidemiology of seasonal influenza after the 2009 influenza pandemic in Africa: a systematic review

**DOI:** 10.4314/ahs.v20i4.5

**Published:** 2020-12

**Authors:** Adamou Lagare, Soatiana Rajatonirina, Jean Testa, Saidou Mamadou

**Affiliations:** 1 Centre de Recherche Médicale et Sanitaire (CERMES), Niamey, Niger; 2 World Health Organization, Regional Office for Africa, Brazzaville, Congo; 3 Université Abdou Moumouni, Niamey, Niger

**Keywords:** Influenza, epidemiology, review, Africa, post pandemic

## Abstract

**Background:**

Influenza infection is a serious public health problem that causes an estimated 3 to 5 million cases and 250,000 deaths worldwide every year. The epidemiology of influenza is well-documented in high- and middle-income countries, however minimal effort had been made to understand the epidemiology, burden and seasonality of influenza in Africa. This study aims to assess the state of knowledge of seasonal influenza epidemiology in Africa and identify potential data gaps for policy formulation following the 2009 pandemic.

**Method:**

We reviewed articles from Africa published into four databases namely: MEDLINE (PubMed), Google Scholar, Cochrane Library and Scientific Research Publishing from 2010 to 2019.

**Results:**

We screened titles and abstracts of 2070 studies of which 311 were selected for full content evaluation and 199 studies were considered. Selected articles varied substantially on the basis of the topics they addressed covering the field of influenza surveillance (n=80); influenza risk factors and co-morbidities (n=15); influenza burden (n=37); influenza vaccination (n=40); influenza and other respiratory pathogens (n=22) and influenza diagnosis (n=5).

**Conclusion:**

Significant progress has been made since the last pandemic in understanding the influenza epidemiology in Africa. However, efforts still remain for most countries to have sufficient data to allow countries to prioritize strategies for influenza prevention and control.

## Introduction

Globally, influenza virus infections result in substantial morbidity, mortality and economic losses every year^1^–^3^. Approximately 10–20% of the world's population are infected with seasonal influenza virus annually, of which 3–5 million are severe cases 4, 5. Young children, pregnant women, the elderly, and persons with underlying medical conditions are at increased risk of influenza-associated illness 6, 7. Recent estimates indicated that 291,243–645,832 seasonal influenza-associated respiratory deaths occur annually globally with the highest mortaity rates observed in sub-Saharan Africa (2•8–16•5 per 100000 individuals) compared to other Regions 8.

The epidemiology of influenza is well-documented in high-income countries, however in Africa fewer data are available 6, 7, 9. A literature review on influenza in sub-Saharan Africa conducted between 1980 and 2009 found that most countries in this region did not have sufficient data on inflenza to prioritize strategies for influenza prevention and control 10.

Nonetheless, after the emergence of the pandemic influenza virus A(H1N1) in 2009 A(H1N1)pdm09 concerted efforts from the World Health Organization (WHO) Member States have led to significant increases in trained personnel and equipped laboratories leading to influenza surveillance expansion, including the capacities to detect and monitor influenza viruses globally^5^,^11^. As a result, several international institutions and governments, in partnership with African countries, invested in the development of epidemiologic and laboratory influenza surveillance capacity on the continent and the African Network of Influenza Surveillance and Epidemiology (ANISE) was formed in 2009 6. In addition, in 2011, WHO initiated a project “Strengthening Influenza Sentinel Surveillance in Africa (SISA)” in 8 African countries to help improve influenza sentinel surveillance, including both epidemiological and virological data collection, and to develop routine national, regional and international reporting mechanisms 12. Since 2009 over 30 countries in Africa have established or expanded influenza surveillance systems 13–21 and 14 countries have received National Influenza Center (NIC) recognition from the WHO 7. Analysis from a regional study in Africa on influenza surveillance revealed that the number of African countries reporting data to WHO's global platform FluNet increased substantially after the pandemic 6.

The burden of influenza has been studied almost exclusively in developed settings, but influenza may have a different pattern in lesser resourced settings such as Africa. It has been suggested that the burden of influenza-associated illness, may be higher in Africa than in other regions due to socio-economic factors, high prevalence of underlying medical conditions, including a heavy burden of HIV and tuberculosis infections, and poor access to care 6, 22. Nonetheless, policies and interventions to mitigate the impact of influenza-associated illness in Africa are lacking 23.

In this study, we aim to assess the state of knowledge gathered from seasonal influenza epidemiology in Africa and identify potential data gaps for policy formulation following the 2009 pandemic.

## Methods

### Literature search Strategy OR data source

In this retrospective inventory, we searched for articles published between 1^st^ January 2010 to 31^st^ December 2019 using four data sources including the U.S. National Library of Medicine (PubMed), Google Scholar, Cochrane Library and Scientific Research Publishing. We considered studies from all African countries, including those belonging to the WHO Regional Office for Africa (AFRO) and WHO Regional Office for the Eastern Mediterranean (EMRO) including Morocco, Tunisia, Egypt, Sudan, Libya and Djibouti 24. However, we did not take in account datarom la Reunion and Mayotte since these two countries are administratively part of the France overseas departments and their data were usually included in national data from France until very recently.

Consistently with the systematic review conducted during 1980–2009 10, we searched for the following terms: (“influenza” AND “Africa”) OR (“acute respiratory infection” AND “Africa”) OR (“influenza” AND “each individual African country”).

### Study selection

We considered studies on influenza surveillance, influenza risk factors and co-morbidities, influenza burden, influenza vaccination or treatment, influenza and other respiratory pathogens, and influenza diagnostic techniques. We searched references of identified studies for additional studies, and we reviewed abstracts and titles of selected studies if they included some aspects of influenza epidemiology consistent with the inclusion criteria mentioned above.

We excluded from this review: studies published before 2010, studies conducted exclusively before 2010 (although published after 2010), studies not presenting data from African countries only, studies reporting a review of literature, studies reporting data on avian influenza only and studies published in other languages than English and French.

### Data abstraction

AL conducted the search to screen and select papers of interest from the four databases and entered the data into a structured Excel database. Considered abstracts from selected papers were reviewed by SR and JT for validation. SR, JT, SM approved all processes for data abstraction and analysis. Any disagreements were resolved after discussion.

For quality assessment of the system, 20% (n=22) of randomly selected articles not considered and all the considered articles (n=199) were reviewed by SR to check for abstraction accuracy and full content evaluation.

### Data analysis

**Figure 1:**
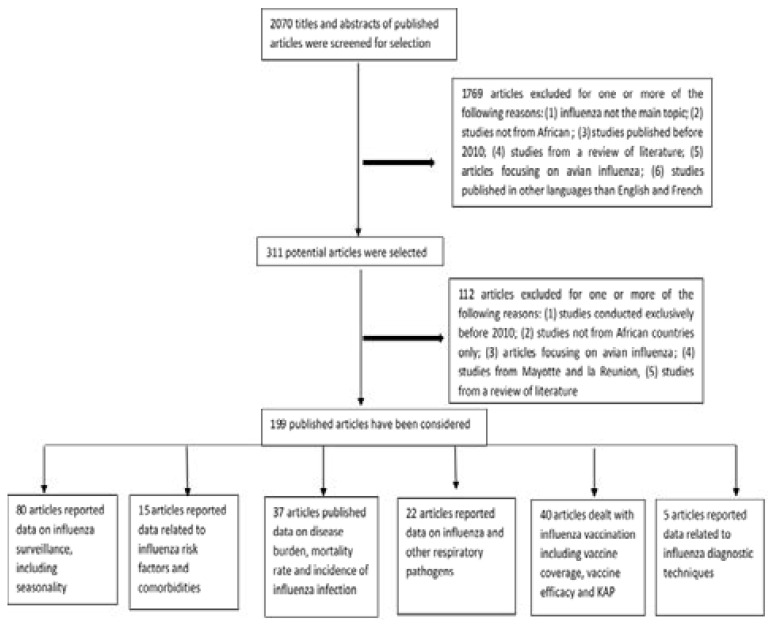
Study design chart

Statistical data analysis was conducted using Excel software. We did a qualitative synthesis of considered articles and grouped the findings of the selected articles according to pre-determined categories. Articles reporting data on influenza circulation, and seasonality including regional or sub regional studies were grouped under the influenza surveillance category. Articles dealing with risk factors associated severe acute respiratory infections (SARI) hospitalization or death and influenza associated with other diseases were grouped uner influenza risk factors and co-morbidities. Articles reporting data on hospitalization and/or mortality rates, incidence and national burden were grouped under the influenza disease burden category. Articles reporting data on the association of influenza and other respiratory pathogens, including viruses and bacteria, were grouped under the influenza and other respiratory pathogens category. The influenza vaccination category consisted of articles reporting studies on vaccine coverage, vaccine efficacy and also knowledge, attitude and practice (KAP) on influenza. Articles reporting studies on influenza diagnostic techniques and assessments were grouped under the influenza diagnostic category (Figure 1).

## Results

### Current status of knowledge

We screened the titles and abstracts of 2070 studies out of which 311 were selected for full content evaluation and of those, 199 studies were considered for analysis. All considered articles were validated according to quality assessment of inclusion criteria and full content evaluation. Selected articles varied substantially on the basis of the topics they addressed: 80 articles reported results of influenza surveillance 13–15, 17–20, 25–91 including 5 regional or sub-regional studies 6, 7, 92–94; 15 articles addressed topics on influenza risk factors and co-morbidities 95–109; 37 articles reported findings on burden of influenza 9, 19, 110–143 including 1 regional article 144; 22 articles reported data on influenza and respiratory pathogens 145–167; 40 articles reported data on influenza vaccination 168–194 including 2 regional studies 195, 196 and 13 KAP 183, 196–207; and 5 articles reported data on influenza diagnostic techniques 208–212. Overall, 30 countries in Africa have articles published on different aspects of influenza epidemiology (Table I).

**Table I:** Distribution of published articles by regions and countries in Africa, 2010–2019

Regions / Countries	Article types (N=199)						

	*INF* *surveillance* (N=80)	*INF risk factor and* *comorbities* (N=15)	*INF burden* (N=37)	*INF and other* *Pathogens* (N=22)	*INF* *vaccination* (N=40)	*INF diagnostic* (N=5)	Total
**North Africa**							
Algeria	1						1
Egypt	2	1	4	2			9
Morocco	3	1			1		5
Tunisia	5	1	2		1	1	10
**West Africa**							
Burkina Faso	3			1			4
Ivory Coast	2				2		4
Gambia				1	1		2
Ghana	3		2	1			6
Mali	1			1	2		4
Niger	2			1			3
Nigeria	1				1		2
Senegal	2		1	2	2		7
Sierra Leone	1						1
Togo	1						1
**Central Africa**							
Cameroon	6			1		1	8
Gabon	1		1	1			3
CAR*	1						1
DRC*	3		1				3
**East Africa**							
Ethiopia	2				1		3
Kenya	10	1	6	2	7	2	28
Madagascar	4		2	2			8
Malawi	1	2	1		3		7
Mozambique	2			1			3
Uganda	6		1				7
Tanzania	1						1
Rwanda	1		1				2
Sudan				1			1
**Southern Africa**							
Angola	1						1
South Africa	9	9	13	5	17	1	55
Zambia			1				1
**Sub-regional studies**	5		1		2		8

Inf: Influenza; CAR: Central Afric a Republic; DRC: De mocratic Republic of Congo (NB: this table does not include cou ntries without any published papers on all influenza aspects betwe en 2010–2019)

**Figure 2:**
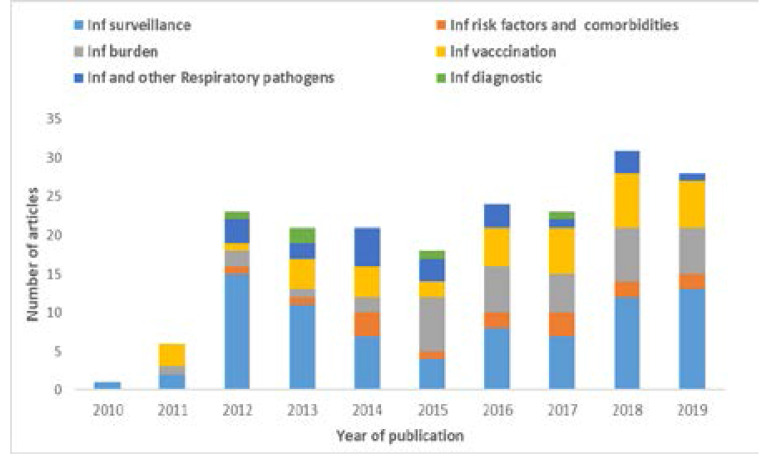
Number and type of published articles per year in Africa, 2010–2019

African countries were grouped according to UNICEF classifications for The State of Africa's Children 2008 based on United Nations regional groupings (https://www.unicef.org/childsurvival/files/SOAC.pdf). Thus, 30 countries from various regions have published influenza data based on our search, with South Africa and Kenya being most represented. The number and types of published articles show a relative increase across the years. From 2010 to 2014 most articles addressed influenza surveillance data, while since 2015 predominant article types reported data on disease burden (including mortality and incidence) and vaccination (Figure 2).

### Influenza surveillance

A total of 27 countries published data on influenza surveillance from 2010–2019. These data covered aspects of influenza detection and seasonality. 21 countries reported influenza surveillance data among inpatients with severe acute respiratory infection (SARI) and outpatients with influenza-like-illness (ILI) 6, 13, 19, 26, 27, 30, 32, 33,36, 37, 40, 45, 46, 51, 55, 61, 64, 69, 70, 72; whereas Gabon, Togo, Burkina Faso, Central Africa Republic and Uganda reported influenza surveillance data among outpatients with ILI only 14, 17, 18, 60, 73, 154.

Globally, the case definition used in most surveillance systems was consistent with that of WHO for influenza-like illness (defined as an acute respiratory infection with measured fever of ≥ 38°C, and cough, with onset within the last 7 or 10 days), or severe acute respiratory infection (defined as an acute respiratory infection with history of fever or measured fever of ≥ 38°C, and cough, with onset within the last 7 or 10 days, and requiring hospitalization) 18, 26, 28, 30, 32, 49. These case definitions were also reported by two regional studies 6, 94. However, other systems used stratified SARI case definition according to various age particularly in younger children 28, 96. Other systems also used modified WHO case definition mainly for severe cases based on the WHO Integrated Management of Childhood Illness guidelines for pneumonia or severe pneumonia 25, 27, 33,34, 39, 96 (Table II). A study from South Africa evaluated the performance of case definition of severe influenza among HIV infected and non-infected patients and found the sensitivity and specificity of the WHO post-2014 case definition more suitable than the previous case definitions irrespective of HIV infection status^85^. Three studies reported results of the evaluation of influenza surveillance systems and found that the influenza surveillance system provided pertinent evidence for public health interventions related to influenza situational awareness although important parameters such as case definition and timely reporting of data should be strengthen to promote prevention interventions especially among the most vulnerable groups^28^, ^81^, ^89^.

**Table II:** Summary of influenza surveillance data in selected Africa countries from 2010–2019

Country	Author	Study period	Enrolled patients	ILI detection rate (%)	SARI detection rate (%)	Total detection rate (%)	Number of sites	Case definition	Method of sampling	Method of detection	Protocol used
Ethiopia	Ayele, et al.	2008–10	176	12	4	7	5	WHO	NP	qPCR	CDC
Ethiopia	Woyessa, et al.	2009–15	4799	27%	3%	20.6	7	WHO	NP/OP	qPCR	CDC
Angola	Cardoso, et al.	2009–11	691	5	6	6	1	WHO	NP/OP	qPCR	CDC
South Africa	Cohen, et al.	2009–12	16,005	-	8	8	SSP	WHO modified	NP/OP	qPCR	CDC
South Africa	Seleka, et al.	2005–14	39,804	40.2	7.9	22	VW/SSP/ILI	WHO modified	NP/OP	qPCR	CDC
Uganda	Cumming, et al.	2010–15	10,142	12.8	8.5	11.2	12	WHO	NP/OP	qPCR	CDC
Uganda	Wabwire, et al.	2008–14	6,628	10.4	-	10.4	5	WHO modified	NP/OP	qPCR	CDC
Nigeria	Dalhatu, et al.	2009–10	2,841	8	5	7.7	4	WHO	NP/OP	qPCR	CDC
Kenya	Emukule, et al.	2007–13	55,192	16.3	8.8	12.2	12	WHO modified	NP/OP	qPCR	CDC
Kenya	Katz, et al.	2007–13	38,775	14.6	9.6	11.4	11	WHO modified	NP/OP	qPCR	CDC
Kenya	Onyango, et al.	2007–10	2002	3.9	4.7	4.9	1	WHO modified	NP	qPCR	Lassaniére
Niger	Halima, et al.	2009–13	2,128	12	6	9.4	8	WHO	NP	qPCR	CDC
Ghana	Jones, et al.	2010–13	1,273	6	8	7	3	WHO modified	NP	qPCR	CDC
Ivory Coast	Kadjo, et al.	2003–10	5,074	19%	-	19%	26	CDC	NP	qPCR/culture	Cambodge
Gabon	Lekana, et al.	2009–11	966	13.6	-	13.6	4	WHO	NP	qPCR	-
CAR	Manirakiza, et al.	2010–15	5,385	8.4	-	8.4	2	WHO	NP/OP	qPCR/PCR	-
Tanzania	Mmbaga, et al.	2008–10	1,794	8.5	7.3	8	5	WHO modified	NP/OP	qPCR	CDC
Tunisia	El Moussi, et al.	2008–11	7,77	-	-	50.8	268	WHO	NP/OP	qPCR	Abott
Madagascar	Orelle, et al.	2009–10	2,303	-	-	49.8	20	WHO	NP/OP	qPCR	CDC
Madagascar	Rajatonirina, et al.	2009–10	750	33.2	-	33.2	24	WHO	NP/OP	qPCR	CDC
Madagascar	Rakotoarisoa, et al.	2009–14	9192	38.9	-	39.9	12	WHO	NP/OP	qPCR	CDC
Malawi	Ho, et al.	2011–13	1126	-	14.5	14.5	1	WHO Modified	NP	qPCR	CDC
Mozambique	Nguenha, et al.	2014–16	1997	-	3.9	3.9	2	WHO	NP/OP	qPCR	CDC
Burkina	Tarnagda, et al.	2010–11	881	6.6	-	6.6	6	WHO	OP	qPCR	CDC
Burkina	Sagna, et al.	2013–15	1392	14.9	-	14.9	2	WHO	OP	qPCR	CDC
Marocco	Rhaffouli, et al.	2009–11	1,183	31	-	31	-	WHO modified	NP	qPCR	Roche
Rwanda	Wane, et al.	2009–10	2,045	14	12	26	6	WHO	NP/OP	qPCR	CDC
Cameroon	Monamele, et al.	2009–15	5,216	-	-	22.2	5	WHO	NP	qPCR	CDC
DRC	Muyembe, et al.	2009–11	4,156	20	16	15	5	WHO	NP/OP	qPCR	CDC
DRC	Kavubga, et al.	2015	2376	-	-	9.1	5	WHO	NP/OP	qPCR	CDC
Togo	Maman, et al.	2010–12	955	24.7	-	24.7	2	WHO	NP/OP	qPCR	CDC

*RCA : Central Africa Republic ; DRC : Democratic Republic of Congo ; SSP : SARI surveillance program ; VW : viral watch, NP : nasopharyngeal swab ; OP : oropharyngeal swab

Influenza surveillance in many African countries was dominated largely by pediatric inclusion of children < 5 years although it aims to target all age categories 6, 14,18, 19, 27, 30, 34, 39, 98. In these systems, the high influenza detection rate was therefore due to infections in this age group 14, 18, 34, 39, although in a few countries the highest influenza positivity rate was observed among children 5–14 years and older adults 17, 25, 27, 32, 53.

The rate of detection of influenza viruses varied considerably according to countries geographical location. It has been reported to be higher in temperate countries ranging from 33% to 50% in Madagascar, Morocco and Tunisia 49, 53, 54, 56, 96. In most sub-Saharan countries influenza detection rate seemed to be relatively low ranging from 6% to 10% in Burkina Faso, Niger, Nigeria, Ghana, Republic of Central Africa, Angola, Tanzania^14^, ^18^, ^30^, ^33^, ^36^, ^45^, ^213^. However, other countries have reported influenza detection rate ranging from 10% to 26% including Kenya, South Africa, Senegal, RDC, Gabon, Uganda, Rwanda and Ethiopia 13, 15, 19, 32, 42, 58, 62, 77.

Importantly, in most countries, influenza was more detected among ILI than SARI cases 19, 27, 32, 33, 58, 77, 96, 98. The lowest influenza detection rate among ILI was 3.9% reported by Onyango et al. in Kenya 52 while the highest rate was 40.2% reported by Seleka et al. in South Africa^58^. Influenza seemed to be less detected among SARI cases ranging from 4% to 12% 27, 31, 62, although studies from Angola, Ghana and Kenya reported a higher rate of detection of influenza SARI compared to ILI cases^30^, ^36^, ^52^ (Table II). A study from Cameroon reported influenza C virus among hospitalized patients apart from seasonal influenza types A and B, although on limited proportion 86.

Influenza testing was conducted on either nasopharyngeal and or oropharyngeal specimens collected from persons presenting for medical care at participating surveillance sentinels sites 14, 16, 17, 27, 28, 32, 39, 48, 81, 89, 92, 96. Most countries were applying a sampling restriction number for SARI and/or ILI at up to 5 cases sampled per week 14, 17, 18, 34, however in other systems, the sampling restriction was only applicable to ILI cases while all SARI cases were collected routinely 27, 28, 33, 39. Laboratory confirmation techniques were based on molecular detection by realtime reverse transcription polymerase chain reaction for the majority of countries^17^–^19^, ^32^–^34^, ^39^, ^49^,^214^. However, countries with national influenza centers used culture and sequencing in addition to the molecular method 48, 71, 75, 82, 84, 96 (Table II).

The seasonality of influenza has been described in many countries and varied considerably. In temperate countries including South Africa, Egypt, Morocco and Tunisia influenza circulation showed regular patterns with one peak of detection during the winter which corresponds to May-September in South Africa 28 similar to other temperate southern hemisphere patterns, and November to April in Egypt, Morocco and Tunisia^49^,^64^, ^96^ similar to temperate northern hemisphere patterns. This has also been described in a regional study reporting the seasonalitof influenza in Africa 6. Influenza activity varied considerably in tropical countries and demonstrated different patterns with double peaks of detection or year-round influenza activity 17–19, 26, 27, 30, 32,33, 39, 51, 213. This has also been reported in two regional studies 6, 7.

### Influenza risk factors and co-morbidities

Although data on risk factors for influenza are key to guide targeted influenza vaccination, few studies have addressed topics on the risk factors for influenza 95–99. Results from these studies reported that underlying medical conditions such as HIV, tuberculosis, diabetes, chronic respiratory diseases, pregnancy and patient's age contribute strongly to influenza associated hospitalization or death. In a case population study between 2009 to 2012 in South Africa, Abandom et al. showed that risk factors for influenza associated SARI hospitalization included history of smoking case-population ratio (CPR) 3.82, HIV infection (CPR 3.61), asthma (CPR 2.45), history of hospital admission in the past 12 months (CPR 2.07), and tuberculosis (CPR 1.85)^95^. Tempia et al. using multivariate analysis on surveillance data from 2012 to 2015 in South Africa reported *Streptococcus pneumoniae* colonization density adjusted odds ratio (aOR) 4.8, malnutrition (aOR, 2.4), prematurity (aOR, 4.8), diabetes (aOR, 7.1), chronic lung diseases (aOR, 10.7), chronic heart diseases (aOR, 9.6), obesity (aOR, 21.3), mine working (aOR,13.8) and pregnancy (aOR, 12.5) as contributing factors for influenza-associated hospitalization 98. Both studies also found an increased risk of hospitalization in those ≤5 years of age (CPR 3.07) and among those 35 years of age and above (CPR 1.23). Moreover, Barakat et al. in Morocco found that influenza cases associated with hypertension (aOR, 28.2), neurological disorders (aOR), obesity (aOR, 7.1), as well as pregnancy (aOR, 2.5) were at increased risk of death 96. Furthermore, Bouneb et al. in Tunisia using logical regression reported that acute respiratory distress syndrome (ARDS) (OR = 27; 95%CI: 3.62–203.78) was the only factor significantly associated with severe outcomes of influenza cases 99.

Influenza associated co-morbidities have been studied nine articles from 3 countries 101–105, 107–109, 134, 215. In two studies on influenza associated respiratory infections in a high HIV Prevalence Setting, Cohen et al. in South Africa found that 44% of patients who tested positive for influenza were also infected with HIV while Ho et al. in Malawi reported a prevalence of 69.6%, therefore supporting the evidence that HIV is an important risk factor for influenza-associated ILI and SARI 100, 102. In other studies, Peterson et al. found that HIV greatly increased the risk of influenza virus-associated SARI in children and von Mollendorf et al. show a potential difference in influenza virus shedding by CD4 count with individuals with lower counts shedding for longer 103, 105. Moreover, in study on household transmission of inflenza from HIV-infected and HIV-uninfected Individuals in South Africa, Cohen et al. found the increased infectiousness of HIV-infected individuals is likely not an important driver of community influenza transmission^108^. Three studies from South Africa reported high correlations between influenza and invasive pneumonia disease or tuberculosis and that tuberculosis influenza co-infection was associated with increased risk of death compared to tuberculosis single infection 101, 109, 215.

### Influenza burden

Overall, 37 articles from 14 countries 9, 82, 110–124, 128–131, 133,135–137, 139–143, 216, 217 including 1 sub-regional paper 144 have reported data on influenza burden, influenza associated mortality, case fatality rate and incidence. Seven papers have reported data on mortality associated with influenza SARI cases 110–112, 116, 118, 120, 138, 139, 144. The estimated incidence of influenza-associated SARI deaths per 100,000 population was highest in children <1 year (20.1, 95%CI 12.1–31.3) and adults aged 45–64 years (10.4,95%CI 8.4–12.9) in South Africa 111 while in Egypt the SARI case fatality rates by influenza virus type were: 50% A/H5N1, 17% INF A and B 120. Also, Emukule et al. found the estimated mean annual rate of influenza associated pneumonia hospitalization was 34 (95% CI 23–48) per 100,000 persons among children in Uganda^138^.

Four articles reported data on mortality associated with influenza and RSV among SARI patients 110, 116, 139, 216. Cohen et al. found that seasonal influenza and RSV allcause mortality rates in South Africa were 23.0 (95% CI 11.0–30.6) and 13.2 (95% CI 6.4–33.8) per 100 000 population annually 110, while Emukule reported 14.1 (95% CI 0.0--93.3) and 17.1 (95% CI 0.0--111.5) per 100,000 person-years respectively for influenza and RSV^116^.

12 articles reported data on influenza national burden associated SARI hospitalization 9, 113, 119, 121, 123, 125, 127–130,135, 141, 142. These rates varied considerably, for example it was estimated at 21 (95% CI 19–23) in Kenya 113, 34.7 (95% CI 25.4–47.7) in Rwanda 127 and 43.9 (95% CI 30.7–57.1) in Zambia 130. However, SARI hospitalization was found to be much higher in children < 5 years from all the studies. The rates per 100,000 individuals were 135 in Ghana 126, 100 in Kenya 216, 128 in Madagascar^141^, 168 in Rwanda 127, 156 in South Africa 100 and 187 in Zambia^130^.

Three studies have estimated rates of influenza-associated ILI and SARI among HIV-infected and HIV-uninfected patients and found influenza-associated SARI and or ILI incidence rate greater among HIV-infected individuals 125, 133, 134. The overall attributable fraction for influenza virus detection to illness was 92.6% for ILI, 87.4% for SARI, and 86.2% for severe chronic respiratory illness (SCRI) in South Africa supporting the evidence that influenza viruses when detected in patients with ILI, SARI, or SCRI are likely attributable to illness^134^. A study from Tunisia estimated proportions of influenza-associated ILI at 3.16% in the total outpatient load 131.

Five articles reported burden data from selected populations in different countries 114, 115, 124, 126, 132. Three studies reported ILI incidence which was 9.9 (95%CI 2.9–33.6) in children < 5 years in Senegal 115 and 3,448 (95% CI 3,727 – 3,898) in Ghana 126 among children <5 years. The other study reported incidence of 24 per 100 person-years among pregnant women in Malawi 114. SARI hospitalization rates in a selected site were reported to be 44 cases per 100 000 person-years (95% CI 39–48) in Egypt 132 and 14.7 (95% CI 9.1- 22.2) in Kenya among persons > 5 years 124.

### Influenza and other respiratory pathogens

A total of 22 papers from 13 countries have reported data on influenza and other respiratory pathogens including both viruses and bacteria 145–167. From these, 13 studies reported data on respiratory pathogens among children < 5 years with SARI 145, 146, 149, 151, 153, 155, 156, 161, 162,165–167, 218, while 9 studied reported data on respiratory pathogens among other age groups 147, 148, 150, 152, 157–160, 163. The detection of respiratory virus varied considerably. In most studies among children <5 years, respiratory syncytial virus was the predominating virus detected in a proportion of 14.1% in Ghana 153 to 81% in Sudan^48^. However, other studies reported rhinovirus as the predominating virus detected in a proportion of 20.5% to 59.1% respectively 151, 155. Two studies also reported respiratory virus detection among ILI cases in children < 5 years 154 and persons > 50 years 147. From these studies adenovirus was the predominant virus detected in a proportion of 17.5% and 22% respectively.

Besides viruses, bacterial pathogens were also reported in many studies as etiological agents of respiratory infections associated with influenza infection. *Streptococcus pneumonia* was the main bacterial pathogen detected (> 50%) and found mostly associated with respiratory viruses including influenza as coinfections 145, 149, 165. Three studies reported that influenza was associated with pneumonia in children < 5 years^145^, ^159^, ^160^.

### *Influenza* vaccination

Influenza vaccine is rarely used in most African countries. However, a total of 40 articles from 12 countries have reported data on influenza vaccine including vaccine efficacy, vaccine coverage and KAP 168–178, 180, 181, 189,190, 192, 194, 219. Vaccine effectiveness and safety was reported in 14 papers 168, 171, 173–179, 181, 189, 190. Three studies have reported the effectiveness of trivalent inactivated influenza vaccine in pregnant women, resulting in protecting their infants against influenza illness 172, 177, 179. However, other studies reported moderate influenza vaccine effectiveness (VE) in preventing medically attended influenza-associated respiratory illnesses in Kenya 171 and South Africa 175, 176. The results from two randomized studies, one from Senegal on live attenuated influenza vaccine against H1N1pdm09 181 and two from South Africa on influenza vaccine efficacy among HIV infected pregnant women reported effective protection among pregnant women and poor efficacy to protect their infants^169^, ^173^.

A regional study on influenza vaccines and antiviral drug availability in Africa estimated a low coverage of the continent 195. From this study, 19/31 (65%) countries reported availability of antiviral drugs for the treatment of influenza while vaccine coverage ranged from < 0.5% to 2% of the population. However, a study from Kenya reported that 64% of health care personnel received monovalent influenza A (H1N1) pandemic 2009 vaccine in 2010 178. Duque et al. reported that although the Northern and Southern hemisphere influenza vaccine formulations have been identical over recent years, the Southern Hemisphere formulation is most widely used in Africa despite its lower production worldwide^195^.

The cost-effectiveness of influenza vaccine has been studied in a Malian paper which estimated the cost of maternal influenza immunization in pregnant woman and found that a maternal influenza immunization program in Mali would cost $857 (95% UI: $188-$2358) per disability-adjusted life year (DALY) saved 180. In another study from South Africa, Biggerstaff et al. found the cost effectiveness of vaccinating the cohort of pregnant women with prioritization had lower societal cost compare to vaccination without prioritization^191^.

Knowledge, attitude and practice (KAP) towards influenza vaccine pointed mainly to the level of education, socio-cultural conspiracy, media access, and logistical challenges as influencers of vaccine coverage in Africa^183^,^193^, ^196^–^201^, ^203^, ^205^–^207^. However, a survey among healthcare workers in Ivory Coast and Kenya revealed that most were willing to accept vaccination if they had adequate informaton on safety and efficacy^202^, ^204^.

### Influenza diagnostic

A total of 5 articles have evaluated diagnostic techniques for influenza detection 157, 208–211. Two studies from Tunisia and South Africa evaluated the use of multiplex rRT-PCR in the identification of respiratory viruses with a sensitivity > 90% and Specificity of 100% thus allowing effective and fast diagnosis of respiratory viral infections 157, 208. Feikin et al. found that serology, along with PCR, can maximize etiologic diagnosis in epidemiologic studies 209. Other studies have evaluated the use of rapid influenza diagnostic tests compared to rRTPCR. Kenmoe et al. in Cameroon found a lower sensitivity (29.4%) of the SD Bioline rapid antigen test 210 while Ndegwa et al. in Kenya found acceptable sensitivity (77.1%) for the Becton Dickinson Veritor™ System Flu A + B rapid influenza diagnostic test (RIDT) 211. Both studies concluded the advantage of using a rapid test for identification of influenza cases with regard to the high specificity (100%) although recommending that negative results be confirmed by rRT-PCR.

## Discussion

Previous reviews of seasonal influenza in Africa published in 2002 and 2010 reported paucity of influenza data before the 2009 pandemic 10, 220. However, since then, significant progress have been made to address some of the critical deficits in knowledge about the epidemiology and burden of seasonal influenza in Africa^7^,^221^.

From our search, 199 published articles from 30 countries have been recorded covering various aspects of influenza surveillance, influenza risk factors and co-morbidities, influenza burden, influenza vaccination, influenza diagnostics, influenza and coinfection with other respiratory pathogens. This significant increase shows the main advances made by the continent since the pandemic as compared to that reported by Gessner et al. from 1980 to 2009^10^. This important progress could be attributed to many interventions and supports from international institutions mainly WHO and CDC Atlanta towards building routine influenza surveillance systems in African countries 6.

While many countries have published data on influenza surveillance system, only few countries have addressed influenza risk factors. Results of these studies clearly reported young and older age, pregnancy and underlying medical conditions such as HIV, obesity, asthma, malnutrition, chronic heart disease etc. as main factors contributing to influenza severity in Africa. These factors were similar in some extent to those reported by studies conducted in other continents, particularly in Europe and USA where influenza infection has been shown to be associated mostly with older age, pregnancy, underlying medical conditions and immune-deficiency^222^, ^223^. Three studies reported that tuberculosis-influenza co-infection was associated with increased risk of death compared to tuberculosis single infection^101^, ^109^, ^215^. These findings were partly confirmed by the results of a recent study on the systematic review of the coinfection of influenza and tuberculosis which found pulmonary tuberculosis (PTB) to be a risk factor for influenza-associated hospitalization 224.

Influenza transmission through various age groups and seasonality have been clearly identified in countries from temperate regions 225, 226, however in many African countries particularly in the sub Saharan region more data need to be gathered in order to define clearly the seasonal pattern of influenza infection 6, 7. In these systems, influenza detection was largely predominated by pediatric inclusion that is children < 5 years 14, 17, 18, 45,127, 165, 227 which limit the capacity to stratify the disease transmission through various age groups. In addition, the relatively low influenza detection rate reported in many countries could be explained by geographical conditions and level of influenza surveillance.

Before the 2009 pandemic, most countries in Africa lack data on influenza burden estimate, making it difficult for policy-makers to decide on how to distribute limited resources 228. Since 2010, nine countries including South Africa, Kenya, Madagascar, Ghana, Senegal, Egypt, Zambia, Rwanda and DRC have published national burden estimate data 110, 111, 113, 115, 119, 121, 127, 130, 135, 137,216. From these studies, the estimated rates (per 100,000 population) of influenza-associated SARI hospitalization among children aged <5 years was 135 in Ghana 126, 100 in Kenya 216, 128 in Madagascar 141, 168 in Rwanda^127^, 156 in South Africa 100 and 187 in Zambia 130. These findings were concordant with those from a global estimate of influenza hospitalization associated respiratory infections among children < 5 years from 1982 to 2012 which reported that Africa has the highest rate of influenza hospitalization estimated at 174 per 100,000 229. Meanwhile, the estimated rates of influenza-associated ILI outpatient consultation per 100,000 population was 1205 in DRC 135, 895 in Ghana 126, 720 in Kenya 230 and 1337 in South Africa 231. These estimates were higher than those reported in a study in the USA 232.

Although important progress has been made by some countries mainly South Africa, Kenya, Senegal and Madagascar efforts still remain for most countries to have sufficient data to allow prioritization of strategies for influenza prevention and control. In addition, there is also lack of influenza economic burden data for all the countries in Africa, although a recent study from South Africa found substantial economic burden of influenza-assciated illness from both a government and a societal perspective estimated at $270.5 million annually^142^. These estimates were consistent with those reported in Bangladesh where the economic burden was estimated at $ 169 million in 2010 233; however lower than that reported from a study in USA which estimated average annual total economic burden of influenza to the healthcare system and society at $11.2 billion 234. Vaccination remains the most efficacious means of mitigating the harmful healthcare and social effects of influenza, however, this practice is not yet implemented in many countries due to behavioral consideration and vaccines costs 188, 200, 204. Therefore, other control measures for prevention of seasonal influenza in the continent such as hygiene measures, use of personal protective equipment and self-quarantine could represent alternative methods (https://www.cdc.gov/flu/prevent/actions-prevent-flu.htm). Influenza vaccines have been shown to be efficient in protecting against influenza infection. However, its coverage is still very low in Africa (<2%) 195, compared to 75% in Europe (ECDC) and 81.1% among children in US (CDC). Pregnant women have a particularly high risk of illness and hospitalization from influenza. Two studies have reported effective protection of influenza vaccine among pregnant women and poor efficacy to protect their infants 169, 173. However, results from a recent review study found that vaccination in a later trimester could benefit both the mother and newborn 235. Indeed, recent estimates of the cost per hospital day averted and the cost per year of life saved by influenza vaccination in South Africa recommended that pregnant women and HIV-infected persons to be prioritized for publicly funded influenza vaccination given available evidence on influenza-associated disease burden 140.

This study presents two main limitations. First, we did not conduct meta-analysis of data which would have allowed qualitative and quantitative assessments using previous studies findings. Second, we only used free access online databases which could have slightly underestimated the number of published papers particularly from the northern African countries where many studies were reported into regional journal which were not indexed.

## Conclusion

This literature review makes an important contribution to knowledge about the epidemiology of seasonal influenza in Africa and that much more is known about influenza in Africa since the last pandemic than ever before. The increase in influenza surveillance in the region and the availability of documented national data make it possible to show the variable contribution of influenza infection across the continent. Indeed, these observed advances in influenza surveillance improve the contribution of the continent to the global influenza surveillance network. Importantly, the development of strategies for planning and response based on achievements and lessons learned could be useful for countries in Africa to prepare for the next pandemic. However, sustainability of the surveillance systems is the main challenges in most countries as it relies almost exclusively on international supports. Therefore, national data on influenza disease burden could be useful for decision makers to assess the public health importance of influenza, to identify high risk groups and regions, to allocate resources efficiently, and to consider the cost-effectiveness of preventive strategies, such as vaccination.
